# The Dissector or Practical and Surgical Anatomy

**Published:** 1851-07

**Authors:** 


ARTICLE II.

*The Dissector or Practical and Surgical Anatomy*:—By ERASMUS WILSON, author of "A System of Human Anatomy," etc. With one hundred and fifteen Illustrations. Edited by PAUL B. GODDARD, M.D. A new and improved edition. pp. 458: 12 mo. Philadelphia: Blanchard & Lea: 1851: (from the publishers through S. C. Griggs & Co., Chicago.

This generally popular work is now improved by the supply of deficiencies created by the advance of investigation, and several new cuts; amongst which, those illustrating the anatomy of the parts concerned in hernia, and of the perinæum, are of the most importance.

The work is so generally known and approved, that it is unnecessary for us to say any thing in its commendation.

